# An updated meta-analysis of optimal medical therapy with or without invasive therapy in patients with stable coronary artery disease

**DOI:** 10.1186/s12872-024-03997-7

**Published:** 2024-07-04

**Authors:** Lei Bi, Yu Geng, Yintang Wang, Siyuan Li, Kuogen Sun, Yiqi Guo, Ou Zhang, Ping Zhang

**Affiliations:** 1 Department of Cardiology, Beijing Tsinghua Changgung Hospital, School of Clinical Medicine, Tsinghua University, NO. 168 Litang Road, Changping District, Beijing, 102218 P. R. China; 2 Beijing Changping District Tiantongyuan North Community Healthcare Center, Beijing, China; 3 Department of Emergency, Tsinghua University Hospital, Beijing, China

**Keywords:** Optimal medical therapy, PCI, CABG, Stable coronary artery disease

## Abstract

**Background:**

The efficacy of optimal medical therapy (OMT) with or without revascularization therapy in patients with stable coronary artery disease (SCAD) remains controversial. We performed a meta-analysis of randomized controlled trials (RCTs) that compared OMT with or without revascularization therapy for SCAD patients.

**Methods:**

Studies were searched in PubMed, EMBASE, and the Cochrane Central Register of Clinical Trials from January 1, 2005, to December 30, 2023. The main efficacy outcome was a composite of all-cause death, myocadiac infarction, revascularization, and cerebrovascular accident. Results were pooled using random effects model and fixed effects model and are presented as odd ratios (ORs) with 95% confidence intervals (CI).

**Results:**

Ten studies involving 12,790 participants were included. The arm of OMT with revascularization compared with OMT alone was associated with decreased risks for MACCE (OR 0.55 [95% CI 0.38–0.80], I²=93%, *P* = 0.002), CV death (OR 0.84 [95% CI 0.73–0.97], I²=36%, *P* = 0.02), revascularization (OR 0.32 [95% CI 0.20–0.50], I²=92%, *P* < 0.001), and MI (OR 0.85 [95% CI 0.76–0.96], I²=45%, *P* = 0.007). While there was no significant difference between OMT with revascularization and OMT alone in the odds of all-cause death (OR 0.94 [95% CI 0.84–1.05], I²=0%, *P* = 0.30).

**Conclusions:**

The current updated meta-analysis of 10 RCTs shows that in patients with SCAD, OMT with revascularization would reduce the risk for MACCE, cardiovascular death, and MI. However, the invasive strategy does not decrease the risks for all-cause mortality when comparing with OMT alone.

**Supplementary Information:**

The online version contains supplementary material available at 10.1186/s12872-024-03997-7.

## Introduction

Coronary artery disease (CAD) is the leading cause of death all over the world, which could cause angina pectoris, acute myocardial infarction (AMI), and ischemic heart failure (HF) [1]. CAD is characterized by the development of atherosclerotic plaques in the epicardial coronary arteries. When atherosclerotic obstruction causes significant flow-limiting, or plaque rupture causes thrombotic vessel occlusion, angina or AMI occurs [2]. Chronic myocardial ischemia caused by stenotic coronary artery or myocardial infarction may further lead to HF and/or death [2], leading that alleviating angina symptoms and preventing AMI or death as the main goals of CAD treatment [1].

Revascularization consisted of percutaneous coronary intervention (PCI) and coronary artery bypass grafting (CABG) have been proved to improve event-free survival in patients with AMI [3, 4]. However, the optimal treatment for patients with stable coronary artery disease (SCAD) are still in controversy. A lot of randomized trials have compared the ability of optimal medical therapy (OMT) and revascularization in achieving aforementioned treatment goals in SCAD patients [5–8]. Most of them found that revascularization provides better symptom relief and improved quality of life compared with OMT [9, 10], but the results whether it can also improve survival or reduce new myocardial infarction are still inconsistent. Therefore, the revascularization always recommends as an adjunct to medical therapy for SCAD patients in guidelines [11].

Accordingly, with the new evidence from long term outcomes of some trials, we sought to conduct this updated meta-analysis to provide a comprehensive assessment of the role of coronary revascularization coupled with OMT compared to OMT alone in patients with SCAD.

## Methods

### Search strategy and data extraction

We carried out the systematic review in accordance with the Preferred Reporting Items for Systematic Reviews and Meta-Analysis (PRISMA) [12] (Table S1 in the Additional file 1). The study protocol has been registered on the International Platform of Registered Systematic Review and Meta-analysis Protocols database (Inplasy protocol: INPLASY202410067). We conducted a search in PubMed, EMBASE, and the Cochrane Central Register of Clinical Trials for RCTs that based on the optimal medical therapy with or without revascularization in patients with SCAD from January 1, 2005, to December 30, 2023. The search strategy was shown in Table S2 in the Additional file 1. The search was complemented by manual search of the reference list of relevant articles and published guideline statements by professional societies.

### Inclusion and exclusion criteria

Inclusion criteria for studies include meeting the following requirements: (1) Patients with SCAD. (2) The patients were treated through optimal medical therapy with or without revascularization therapy. (3) Outcomes indicators: All-cause death, cardiovascular (CV) death, myocadiac infarction, revascularization. We excluded studies that enrolled patients < 18 years old; did not have enough data to extract, such as the summary of some meetings, literature materials such as reviews and pharmacological introductions; non-randomized trials, including observational studies, reviews, and meta-analysis; non-English language manuscripts; trials not in humans; and also studies before 2000 in which had percutaneous transluminal coronary angioplasty or balloon angioplasty as the primary means of intervention, as they did not reflect the current standard of care.

The protocol was drafted by three authors (Lei Bi, Yu Geng, and Yintang Wang) and reviewed by all co-authors. EndNote (X9 version) software was selected for document management, two investigators (Lei Bi and Yu Geng) independently evaluated the eligibility of the identified items. Potential discrepancies were discussed with the senior author (Ping Zhang).

### Outcomes

The primary efficacy outcome was a composite of major cardiac and cerebrovascular events (MACCE), including all-cause death, myocardial infarction (MI), revascularization, and cerebrovascular accident. Other efficacy outcomes were all-cause death, CV death, MI, revascularization, hospitalization, and cerebrovascular accident. Definitions in individual trials were reviewed, and a harmonizing definition was used across the trials to the extent (Table S3 in the Additional file 1). We used the Cochrane Collaboration criteria to determine the risk of bias for each included study.

### Statistical analysis

Revman5.3 were used for meta-analysis. Data that met homogeneity (*P* > 0.10 and I^2^ ≤ 50%) through heterogeneity test were meta-analyzed using fixed effect model. If homogeneity (*P* ≤ 0.10 or I^2^ > 50%) was not met or heterogeneity cannot be ruled out, random effect model was used to combine effects [13]. For the discontinuous outcomes, odds ratio (OR) estimates with the related 95% confidence intervals (CIs) were used. A *P* value < 0.05 was considered statistically significant. And Mantel-Haenszel (MH) was used for between study variance estimation.

### Subgroup and sensitivity analyses

The treatment efficacy of OMT therapy with or without revascularization was explored in patients with SCAD. The revascularization treatment strategy varies in different studies. Additional subgroup analyses were used to compare the efficacy results between PCI or CABG versus OMT of the SCAD patients. R software (version 4.2.2) was used to investigate the influence of a single study on the overall pooled estimate of each predefined outcome.

## Results

**Fig. 1:**
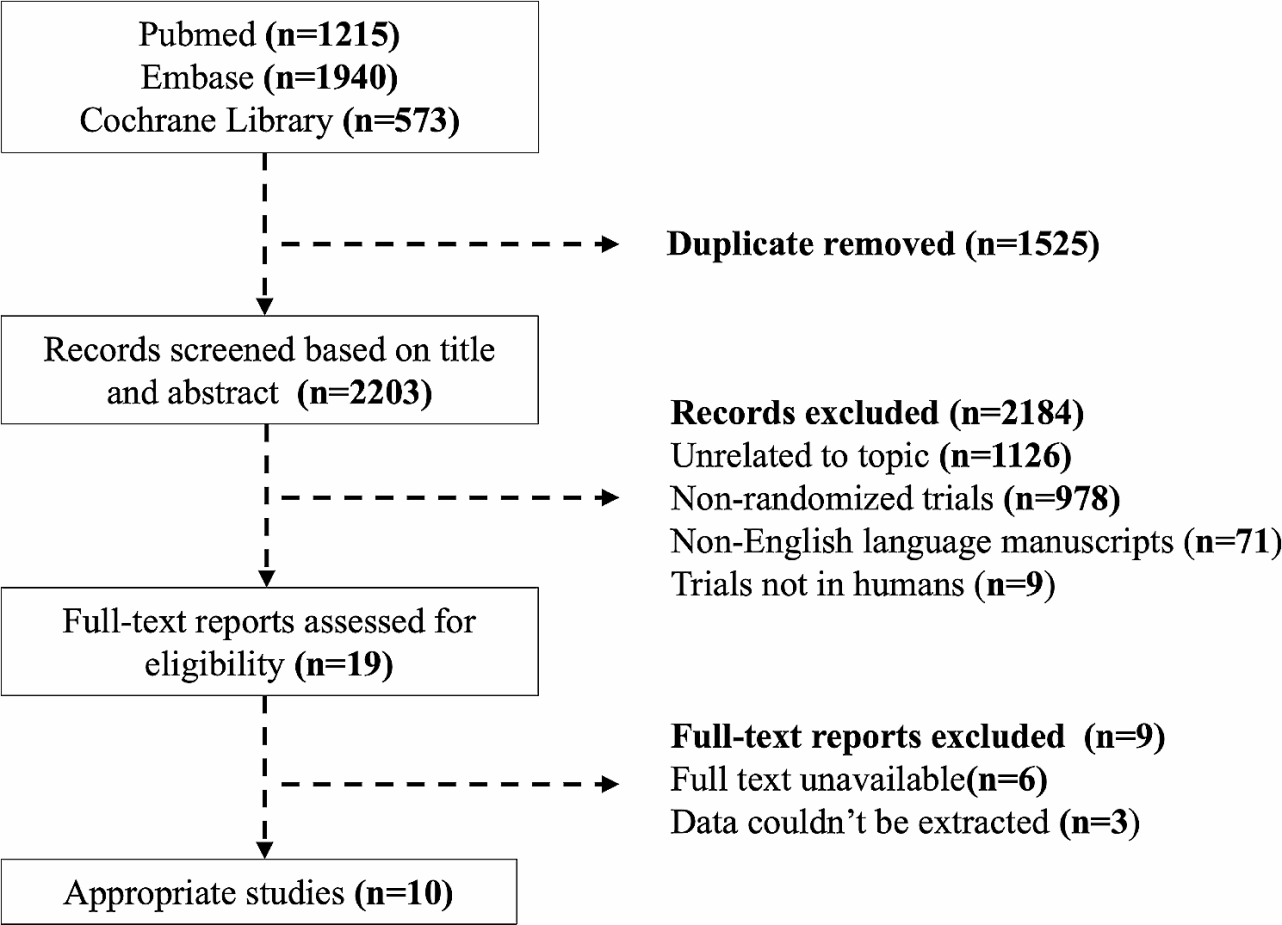
Flow chart of search and study selection process

The flow chart (Fig. 1) summarizes the search and study selection process. A total of 3,728 studies were identified through the search in PubMed, Cochrane Central Register for Controlled Trials, and EMBASE, of which 1,525 were excluded due to duplication. Then, 2,203 irrelevant studies were also excluded after reading the titles and abstracts. The remaining 19 studies were assessed by reading the full texts. Among them, data from 10 RCTs evaluating the efficacy of the optimal medical therapy with or without revascularization in patients with SCAD were included.

In the end, 10 RCTs involving 12,790 patients, comprising 6,497 patients with revascularization and 6,293 patients with OMT alone, were included in the analysis (Table 1). These RCTs include ISCHEMIA [14], ISCHEMIA-CKD [15], ISCHEMIA-EXTEND [16], ISCHEMIA-post hoc [17], BARI 2D [18], MASS II [7], and TIME [5] (trials on OMT with or without PCI and/or CABG), COURAGE [6], FAME2 [19], and JASP [20] (trials on OMT with or without PCI). No differences were observed in terms of the proportion of patients lost to follow up between the groups across trials. As the ISCHEMIA, ISCHEMIA-EXTEND, and ISCHEMIA-post hoc trials included the same patients, the most appropriate one was used in analysis for corresponding outcomes.

**Table 1:** Design and outcomes of the studies included in the current Meta-analysis

Num	Study/Year	Treatment strategy	Revascularization assignments		Patient demographics and comorbidities						The primary outcomes
			Revasc, n	OMT, n	Sample size, n	Mean age, years	Men, %	HTN, %	DM, %	Follow up	
1	FAME2/2008	PCI	447	441	888	64	78.2	77.7	27.0	5y	The primary end point was a composite of death, myocardial infarction, or urgent revascularization.
2	TIME/2004	PCI	140	142	282	80	58.2	60.6	21.6	1y	Major adverse cardiac events (death, nonfatal myocardial infarction, or hospitalization for acute coronary syndrome) after 1 year.
3	BARI 2D/2009	PCI/CABG	1176	1192	2368	62	70.4	NA	NA	5.3y	Worsening angina, freedom from angina, occurrence of new angina, and subsequent coronary revascularization.
4	MASS II/2010	PCI/CABG	408	203	611	60	69.2	29.3	14.4	5y	The primary end point was the incidence of overall mortality, Q-wave myocardial infarction, or refractory angina requiring revascularization.
5	COURAGE/2007	PCI	1149	1138	2287	62	85.1	66.5	33.5	4.6y	The primary outcome was a composite of death from any cause and nonfatal myocardial infarction.
6	JASP /2008	PCI	188	191	379	64	74.2	63.3	40.1	3.3y	The primary end points were death (total death, cardiac death, and sudden death), ACS (AMI or UAP), cerebrovascular accidents (CVA; cerebral infarction or cerebral hemorrhage), and emergency hospitalization.
7	ISCHEMIA/2023	PCI/CABG	2588	2591	5179	64	77.4	73.4	41.8	3.2y	The primary outcome was all-cause, cardiovascular, and non-cardiovascular mortality
8	ISCHEMIA-CKD/2020	PCI/CABG	388	389	777	63	68.9	92.0	57.1	2.2y	The primary outcome was a composite of death or nonfatal myocardial infarction.
9	ISCHEMIA-EXTEND/2023	PCI/CABG	2418	2407	4825	64	77.0	73.0	42.0	5.7y	The primary outcome was all-cause, cardiovascular, and non-cardiovascular mortality
10	ISCHEMIA-post hot/2023	PCI/CABG	2012	2591	4603	64	77.4	73.4	42.2	3.2y	The primary outcome was the composite of death from cardiovascular causes, myocardial infarction, or hospitalization for unstable angina, heart failure, or resuscitated cardiac arrest.

AMI = acute myocardial infarction; CABG = coronary-artery-bypass-grafting; CVA = cerebrovascular accidents; DM = diabetes mellitus; HTN = hypertension; PCI = percutaneous coronary intervention; UAP = unstable angina pectoris

### Clinical outcomes

**Fig. 2:**
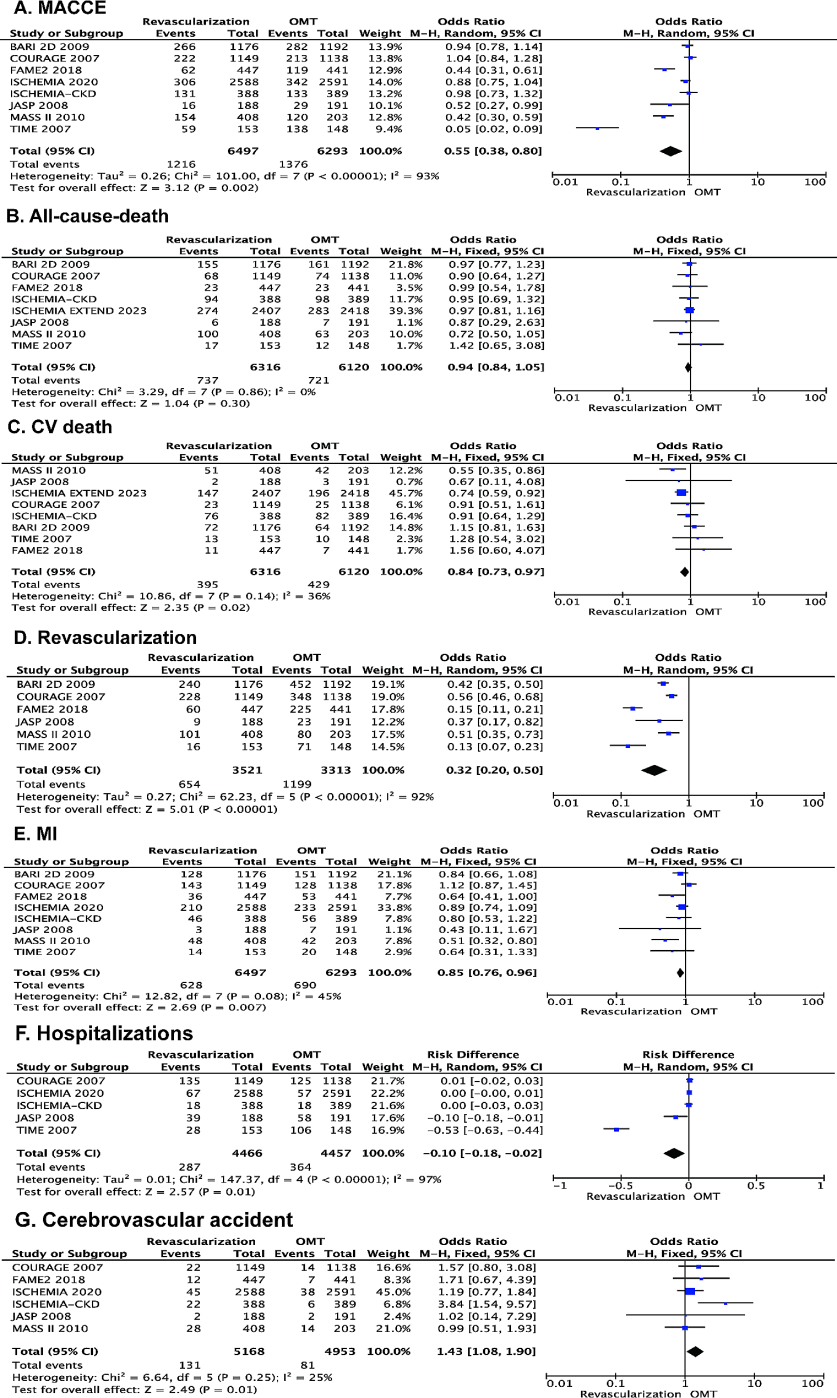
Forest plot of pooled odds ratio (OR) comparing OMT with revascularization versus OMT alone for the efficacy outcomes. **A**: MACCE; **B**: all-cause death; **C**: CV death; **D**: revascularization; **E**: MI; **F**: hospitalizations; **G**: cerebrovascular accident

The MACCE was infrequent and occurred in 1,216 patients receiving OMT with revascularization and 1,376 patients receiving OMT alone. The OMT with revascularization compared with OMT alone was associated with decreased risk for MACCE (OR 0.55 [95% CI 0.38–0.80], I²=93%, *P* = 0.002) (Fig. 2A). Overall, 1,458 cases of all-cause death were reported across the 8 studies, equally split among the 2 therapeutic groups. While there was no significant difference between OMT with revascularization and OMT alone in the odds of all-cause death (OR 0.94 [95% CI 0.84–1.05], I²=0%, *P* = 0.30) (Fig. 2B). The risk for CV death (OR 0.84 [95% CI 0.73–0.97], I²=36%, *P* = 0.02) (Fig. 2C), revascularization (OR 0.32 [95% CI 0.20–0.50], I²=92%, *P* < 0.001) (Fig. 2D), MI (OR 0.85 [95% CI 0.76–0.96], I²=45%, *P* = 0.007) (Fig. 2E), and hospitalizations (OR -0.10 [95% CI -0.18– -0.02], I²=97%, *P* = 0.01) (Fig. 2F) was decreased in the patients receiving OMT with revascularization compared with OMT alone. However, comparing to the OMT alone arm, OMT with revascularization had a significant increase in the risk for cerebrovascular accident (OR 1.43 [95% CI 1.08–1.90], I²=25%, *P* = 0.01) (Fig. 2G).

### Subgroup analysis

We performed a pre-assigned subgroup analysis for patients with different revascularization strategy: 10,043 patients were receiving OMT with PCI versus OMT alone (4,287 were assigned to OMT with PCI and 5,756 to OMT alone) and 5,079 patients were receiving OMT with CABG versus OMT alone (1,093 were assigned to OMT with CABG and 3,986 to OMT alone). The arm of OMT with PCI or OMT with CABG compared to OMT alone was associated with a decreased risk for MACCE (OR 0.63 [95% CI 0.44–0.88], I²=84%, *P* = 0.008) and (OR 0.79 [95% CI 0.63–1.00], I²=96%, *P* = 0.05), respectively; and revascularization (OR 0.46 [95% CI 0.26–0.81] I²=94%, *P* = 0.008) and (OR 0.13 [95% CI 0.09–0.18], I²=0%, *P* < 0.001), respectively (Figure S1 & S2 in the Additional file 1). In addition, the arm of OMT with PCI compared with OMT alone was also associated with decreased risk for CV death (OR 0.81 [95% CI 0.66–1.00], I²=6%, *P* = 0.05) and MI (OR 0.79 [95% CI 0.63–1.00], I²=58%, *P* = 0.05) (Figure S2 in the Additional file 1). However, there was no significant difference between receiving OMT with PCI and OMT alone in the risk for all-cause death (OR 0.89 [95% CI 0.74–1.06], I²=0%, *P* = 0.19) and cerebrovascular accident (OR 1.27 [95% CI 0.82–1.96], I²=0%, *P* = 0.29) (Figure S1 in the Additional file 1). Similarly, the arm of OMT with CABG compared to OMT alone had no significant change in the risk for all-cause death (OR 0.92 [95% CI 0.71–1.20], I²=31%, *P* = 0.54), CV death (OR 0.82 [95% CI 0.44–1.54], I²=78%, *P* = 0.55), and MI (OR 0.75 [95% CI 0.39–1.45], I²=87%, *P* = 0.40) (Figure S2 in the Additional file 1).

### Sensitivity analysis

We used R software to investigate the influence of a single study on the overall pooled estimation of each predefined outcome (the primary efficacy outcome, all-cause death, CV death, MI, and revascularization). We found that the removal of any one study did not affect the results overall, while in terms of MACCE and cerebrovascular accidents, may have been influenced by a single experiment, which may have highlighted the reduced power of these results.

### Risk of bias and quality assessment of outcomes

The results of the risk of bias assessment with the RoB2 of randomized control trials are summarized in Figure S4 in the Additional file 1. Four studies were considered at low risk for overall risk of bias.

## Discussion

In this updated meta-analysis of 10 RCTs which contains 12,790 patients, we found that in patients with SCAD, comparing with OMT alone, revascularization with OMT would reduce the risk for MACCE, cardiovascular death, and MI. Invasive therapy is also associated with a lower rate of revascularization and recurrent hospitalization. However, invasive therapy does not decrease the risks for all-cause mortality. The aforementioned benefit is mainly driven by the strategy of PCI therapy with OMT, and it should be mentioned that the trials included in our meta-analysis have PCI as the predominant means of revascularization, except for BARI 2D, MASS-II, and ISCHEMIA trial in which a significant proportion of patients underwent CABG.

Our finding of a lower risk for the primary efficacy outcomes of MACCE in revascularization with OMT arm is predominantly driven by FAME 2 [19], MASS II [7], and TIME data [5]. The 5-year follow-up data from FAME 2 was published in 2018 [19], and it reported that a coronary fractional flow reserve (FFR) guided PCI led to a significantly lower rate of the prespecified primary composite end point of death, MI, or urgent revascularization than medical therapy alone. In addition, intracardiac imaging by utilizing intravascular ultrasound (IVUS) or optical coherence tomography (OCT) in guiding PCI has consistently shown to reduce major adverse cardiovascular events (CV death, target lesion–related MI, or ischemia-driven target lesion revascularization) [21]. These findings were also in consistent with the results shown in a meta-analysis of 31 studies with 17,882 patients [22]. The current guidelines recommend that revascularization be considered in patients with SCAD when signs of reversible myocardial ischemia are present [11, 23, 24]. Findings in aforementioned studies indicate that revascularization guided by an intravascular technique estimation of the target lesions might be more benefit for the target patients.

A plethora of researches have addressed the potential of revascularization to improve survival and to reduce MIs in patients with stable CAD, and from 2000s, several RCTs were conducted to provide more robust evidence in this field [5–8, 19, 20]. However, almost none of them found the relationship between lower risk for death or MI and revascularization in addition to OMT, except for relief of anginal symptom. Trials before The International Study of Comparative Health Effectiveness with Medical and Invasive Approaches (ISCHEMIA) enrolled patients with milder levels of ischemia, which may be one of the postulations for the negative results. ISCHEMIA was designed to determine the effect of revascularization added to medical therapy in patients with stable CAD and moderate or severe ischemia, for whom an invasive strategy might have been most beneficial [14]. Although the ISCHEMIA trial also failed to find the benefit of revascularization in survival improvement, it found a lower incidence of spontaneous MIs on long-term follow-up in the invasive strategy arm than among those in the conservative-strategy group. This finding indicates that there might be a long-term benefit of revascularization for stable CAD patients. Recently, the results of extended follow-up for mortality of ISCHEMIA trial have been published [16]. With a median follow-up of 5.7 years, the study showed that there was a lower 7-year rate of cardiovascular mortality with an initial invasive strategy, but a higher 7-year rate of non-cardiovascular mortality compared with the conservative strategy, which result in no net treatment difference in all-cause mortality.

Our meta-analysis contains the updated results of extended follow-up of ISCHEMIA trial [16], and all the included trials predominantly reflected the contemporary medical practices in both the medical and the invasive arms, and the data in the analysis based on the longest follow up data available for each trial [5–8, 14−17, 19, 20]. Several meta-analyses aiming at exploring the more beneficial therapy strategy for patients with stable CAD have been published. In meta-analysis published by Bangalore et al., in which 14 RCTs with 14,877 patients were included with a weighted mean follow up of 4.5 years, no difference in mortality was found between medical therapy and revascularization, but a reduced nonprocedural MI in the invasive therapy arm [25]. However, trials included in this meta-analysis were much older and balloon angioplasty was the predominant means of intervention. A recently published meta-analysis conducted by Aviral et al. was similar to the current analysis [26]. It contained 7 RCTs with 12,013 patients and reported that there was no statistically significant difference in primary outcome of all-cause mortality between either arm, but statistically significant lower rates of MACCE (death, MI or stroke), cardiovascular death, and MI in the revascularization arm comparing to conservative arm. Our results are consistent with the analysis by Aviral et al., for the similar including criteria of trials, and in addition, we update the results of extended follow-up for mortality of ISCHEMIA which provides a significant long-term improvement in cardiovascular mortality.

Our finding of lower incidence of revascularization in the invasive with OMT arm are consistent with prior randomized trials of revascularization versus medical therapy alone [5–8, 19, 20]. And the finding of lower incidence of MI in the invasive with OMT arm which is predominantly driven by ISCHEMIA [14], MASS II [7] and FAME2 [19] data, is also consistent with the meta-analysis by Aviral et al. [26], because of the choice to include primary definition of MI in the ISCHEMIA trial [14].

Nevertheless, there are some scenarios in the practice need further discuss. When encountering equal percentages of stenosis in multiple vessels, intravascular technique estimation (FFR, IVUS, and OCT) of the target lesions is crucial. When encountering stenoses with an FFR of 0.81, in such borderline scenario, comprehensive assessment based on symptoms (e.g. assessment of frequency of angina and quality of life), risk factors, or more tools (echocardiography, instantaneous wave-free ratio (iwFR), myocardial contrast echocardiography, late gadolinium enhancement cardiac magnetic resonance, et al.) to estimate myocardial viability or functionally significant stenosis maybe beneficial [1, 11]. Meanwhile, the individual risk-benefit ratio should always be evaluated and revascularization considered only if the expected benefit outweighs its potential risk. Based on thorough assessment of the extent and severity of CAD as well as the presence of associated comorbidities, the aspect of shared decision-making is crucial. Full information must be given to the patient about the anticipated advantages and disadvantages of the two strategies, including the dual antiplatelet therapy related bleeding risks, contrast-induced nephropathy, or procedural complications, and multidisciplinary decision-making maybe required in some scenarios.

### Limitations

Some limitations should be taken into account. First, we did not have access to individual patient data; and the definitions of the primary endpoints of MACCE and the diagnostic method to detect ischemia varied across trials included in this meta-analysis. Second, the findings do not apply to patients with clinically significant left main CAD, low ejection fraction, acute coronary syndrome, or those with class III or IV heart failure. Third, although the aim of our meta-analysis was to assess the benefit of revascularization for stable CAD, the trials included in our meta-analysis have PCI as the predominant means of revascularization, given less patients underwent CABG. But the subgroup analysis also shows lower risk for MACCE and revascularization in patients received CABG and OMT.

## Conclusions

The current updated meta-analysis of 10 RCTs shows that in patients with SCAD, OMT with revascularization would reduce the risk for MACCE, cardiovascular death, and MI. However, the invasive strategy does not decrease the risks for all-cause mortality when comparing with OMT alone.

### Electronic supplementary material

Below is the link to the electronic supplementary material.

Supplementary Material 1

## Data Availability

Data that support the findings of this study are available in the original manuscript of the 10 included RCTs which all can be searched in PubMed. These RCTs include ISCHEMIA, ISCHEMIA-CKD, ISCHEMIA-EXTEND, ISCHEMIA-post hoc, BARI 2D, MASS II, TIME, COURAGE, FAME2, and JASP.

## Abbreviations

AMI: Acute myocardial infarction
CABG: Coronary artery bypass grafting
CAD: Coronary artery disease
CIs: Confidence intervals
CV: Cardiovascular
FFR: Fractional flow reserve
HF: Heart failure
IVUS: Intravenous ultrasound
MACCE: Major cardiac and cerebrovascular events
OCT: Optical coherence tomography
OMT: Optimal medical therapy
OR: Odds ratio
PCI: Percutaneous coronary intervention
RCT: Randomized controlled trials
SCAD: Stable coronary artery disease
