# Supplementary material for: An updated meta-analysis of optimal medical therapy with or without invasive therapy in patients with stable coronary artery disease
Source: BMC Cardiovasc Disord. 2024 Jul 4;24:335. doi: 10.1186/s12872-024-03997-7 (PMC11223390; doi:10.1186/s12872-024-03997-7)
Supplement: Supplementary file 1 — Supplementary Material 1 [file 12872_2024_3997_MOESM1_ESM.docx]

**Supplemental material**

**1. Table S1: PRISMA checklist**

**2. Table S2: Search strategy**

**3. Table S3: Definitions of clinical outcomes**

**4.** **Figure S1: Subgroup analysis of efficacy outcomes between PCI and OMT.**

**5. Figure S2: Subgroup analysis of efficacy outcomes between CABG and OMT.**

**6. Figure S3: Sensitivity analysis of efficacy outcomes comparisons.**

**7. Figure S4: Risk of bias and quality assessment of outcomes**

**Table S1. PRISMA checklist**

| **Section/topic** | **#** | **Checklist item** | **Reported on page #** |
| --- | --- | --- | --- |
| **TITLE** | | |  |
| Title | 1 | Identify the report as a systematic review, meta-analysis, or both. | 1 |
| **ABSTRACT** | | |  |
| Structured summary | 2 | Provide a structured summary including, as applicable: background; objectives; data sources; study eligibility criteria, participants, and interventions; study appraisal and synthesis methods; results; limitations; conclusions and implications of key findings; systematic review registration number. | 3 |
| **INTRODUCTION** | | |  |
| Rationale | 3 | Describe the rationale for the review in the context of what is already known. | 5 |
| Objectives | 4 | Provide an explicit statement of questions being addressed with reference to participants, interventions, comparisons, outcomes, and study design (PICOS). | 5 |
| **METHODS** | | |  |
| Protocol and registration | 5 | Indicate if a review protocol exists, if and where it can be accessed (e.g., Web address), and, if available, provide registration information including registration number. | 6 |
| Eligibility criteria | 6 | Specify study characteristics (e.g., PICOS, length of follow-up) and report characteristics (e.g., years considered, language, publication status) used as criteria for eligibility, giving rationale. | 6 |
| Information sources | 7 | Describe all information sources (e.g., databases with dates of coverage, contact with study authors to identify additional studies) in the search and date last searched. | 6 |
| Search | 8 | Present full electronic search strategy for at least one database, including any limits used, such that it could be repeated. | Supplemental material |
| Study selection | 9 | State the process for selecting studies (i.e., screening, eligibility, included in systematic review, and, if applicable, included in the meta-analysis). | 7 |
| Data collection process | 10 | Describe method of data extraction from reports (e.g., piloted forms, independently, in duplicate) and any processes for obtaining and confirming data from investigators. | 6 |
| Data items | 11 | List and define all variables for which data were sought (e.g., PICOS, funding sources) and any assumptions and simplifications made. | 6 |
| Risk of bias in individual studies | 12 | Describe methods used for assessing risk of bias of individual studies (including specification of whether this was done at the study or outcome level), and how this information is to be used in any data synthesis. | 6-7 |
| Summary measures | 13 | State the principal summary measures (e.g., risk ratio, difference in means). | 6-7 |
| Synthesis of results | 14 | Describe the methods of handling data and combining results of studies, if done, including measures of consistency (e.g., I^2^) for each meta-analysis. | 6-7 |

| Risk of bias across studies | 15 | Specify any assessment of risk of bias that may affect the cumulative evidence (e.g., publication bias, selective reporting within studies). | 6-7 |
| --- | --- | --- | --- |
| Additional analyses | 16 | Describe methods of additional analyses (e.g., sensitivity or subgroup analyses, meta-regression), if done, indicating which were pre-specified. | 7-8 |
| **RESULTS** | | |  |
| Study selection | 17 | Give numbers of studies screened, assessed for eligibility, and included in the review, with reasons for exclusions at each stage, ideally with a flow diagram. | Supplemental Figure 1 |
| Study characteristics | 18 | For each study, present characteristics for which data were extracted (e.g., study size, PICOS, follow-up period) and provide the citations. | Table 1 |
| Risk of bias within studies | 19 | Present data on risk of bias of each study and, if available, any outcome level assessment (see item 12). | Supplement Figure S4 |
| Results of individual studies | 20 | For all outcomes considered (benefits or harms), present, for each study: (a) simple summary data for each intervention group (b) effect estimates and confidence intervals, ideally with a forest plot. | 8-10 |
| Synthesis of results | 21 | Present results of each meta-analysis done, including confidence intervals and measures of consistency. | 8-10 |
| Risk of bias across studies | 22 | Present results of any assessment of risk of bias across studies (see Item 15). | Supplement Figure S4 |
| Additional analysis | 23 | Give results of additional analyses, if done (e.g., sensitivity or subgroup analyses, meta-regression [see Item 16]). | 8-10 |
| **DISCUSSION** | | |  |
| Summary of evidence | 24 | Summarize the main findings including the strength of evidence for each main outcome; consider their relevance to key groups (e.g., healthcare providers, users, and policy makers). | 11-13 |
| Limitations | 25 | Discuss limitations at study and outcome level (e.g., risk of bias), and at review-level (e.g., incomplete retrieval of identified research, reporting bias). | 14 |
| Conclusions | 26 | Provide a general interpretation of the results in the context of other evidence, and implications for future research. | 14 |
| **FUNDING** | | |  |
| Funding | 27 | Describe sources of funding for the systematic review and other support (e.g., supply of data); role of funders for the systematic review. | 16 |

**Table S2. Search strategy**

| **Electronic database** | **Search strategy** |
| --- | --- |
| PubMed (NCBI) | ((("stable coronary artery disease"[Title/Abstract] OR "stable angina"[Title/Abstract] OR "angina"[Title/Abstract])) AND (("medical therapy" [Title/Abstract] OR "conservative management"[Title/Abstract] OR "conservative strategy"[Title/Abstract])) AND (("PCI"[Title/Abstract] OR "revascularization" [Title/Abstract] OR "CABG"[Title/Abstract] OR "surgery" [Title/Abstract]))) |
| Embase | #1 (‘stable coronary artery disease’:ab,ti OR ‘stable angina’:ab,ti OR ‘angina’:ab,ti)  #2 (‘medical therapy’:ab,ti OR ‘conservative management’:ab,ti OR ‘conservative strategy’:ab,ti)  #3 (‘PCI’:ab,ti OR ‘revascularization’:ab,ti OR ‘CABG’:ab,ti OR ‘surgery’:ab,ti)  #4 #1 AND #2 AND #3 |
| Cochrane | (“stable coronary artery disease” OR “stable angina” OR “angina”) AND (“medical therapy” OR “conservative management” OR “conservative strategy”) AND (“PCI” OR “revascularization” OR “CABG” OR “surgery”) |

**Table S3. Definitions of clinical outcomes**

| **The primary efficacy outcomes** | A composite of death, myocardial infarction, urgent revascularization, or stroke. |
| --- | --- |
| **Other efficacy outcomes** | All-cause death: All the deaths that occur in the study population, regardless of the cause. |
|  | CV death: Cardiovascular deaths are defined as all deaths excluding those for which the principal and underlying cause is solely non-cardiovascular. Any death for which a cardiovascular contributing cause is suspected will also be considered a cardiovascular death. |
|  | Myocadiac infarction: Based upon the Universal Definition of MI, but relies upon site-reported MI decision limits for troponin (which may or may not be the same as the manufacturer 99%URL), and has selected unique marker criteria for MI after PCI or CABG  (Type 4a, 5). |
|  | Revascularization: Urgent revascularization was defined as any unplanned hospital admission that was due to symptoms that led to revascularization during the same hospitalization. Subsequent coronary revascularizations were the first PCI or CABG done during the follow-up in the participants randomized to the OMT strategy or the first additional revascularization done in participants randomized to the revascularization s strategy. |
|  | Hospitalizations: Including hospitalization for acute coronary syndrome, emergency hospitalization, or hospitalization for unstable angina, heart failure, or resuscitated cardiac arrest. |
|  | Cerebrovascular accident: Defined as the rapid onset of a new neurologic deficit attributed to an obstruction in cerebral blood flow and/or cerebral hemorrhage with no apparent non-vascular cause (eg. trauma, tumor, or infection). |

**
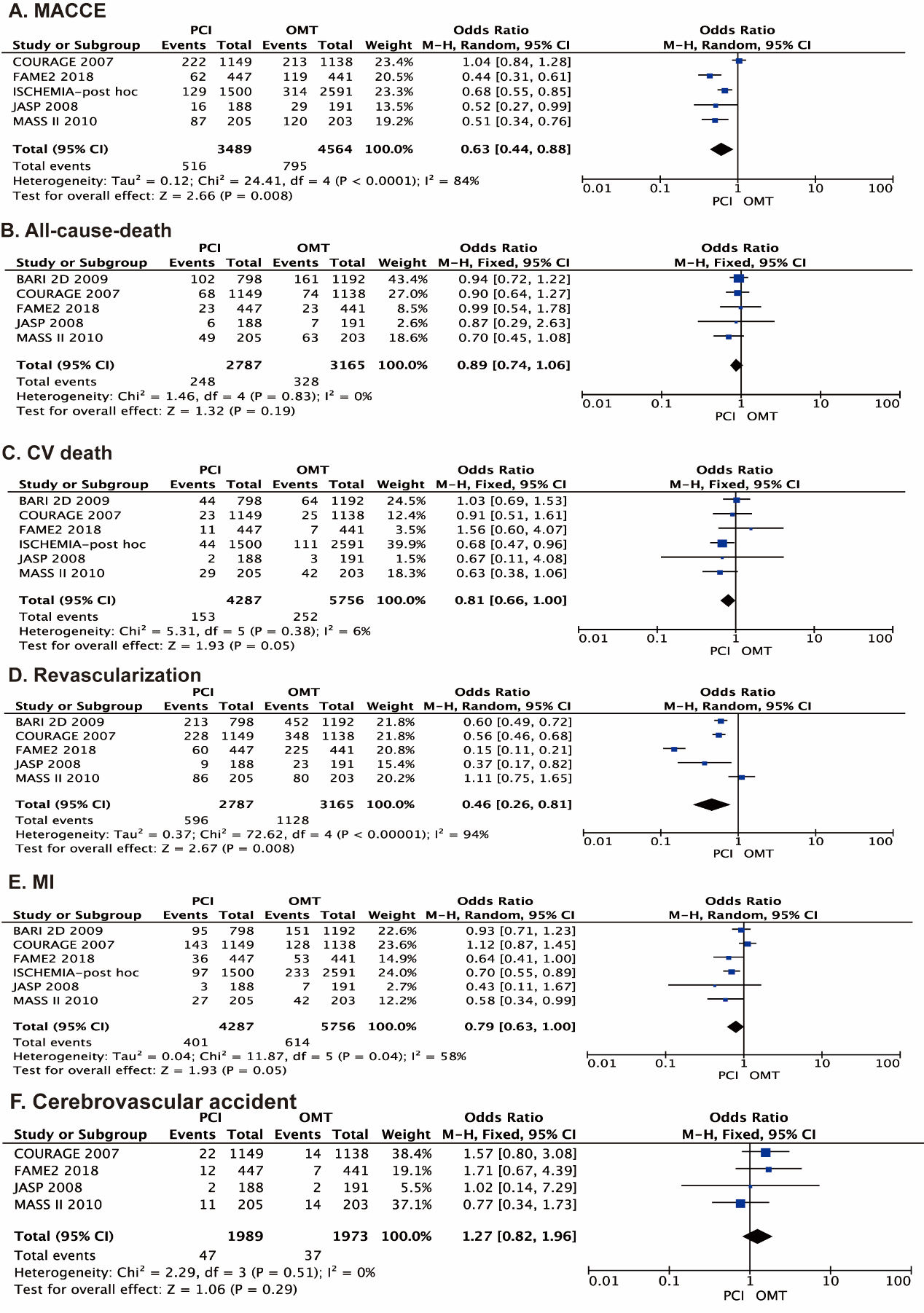
Figure S1:** **Subgroup analysis of** **efficacy outcomes between PCI and OMT.**

**
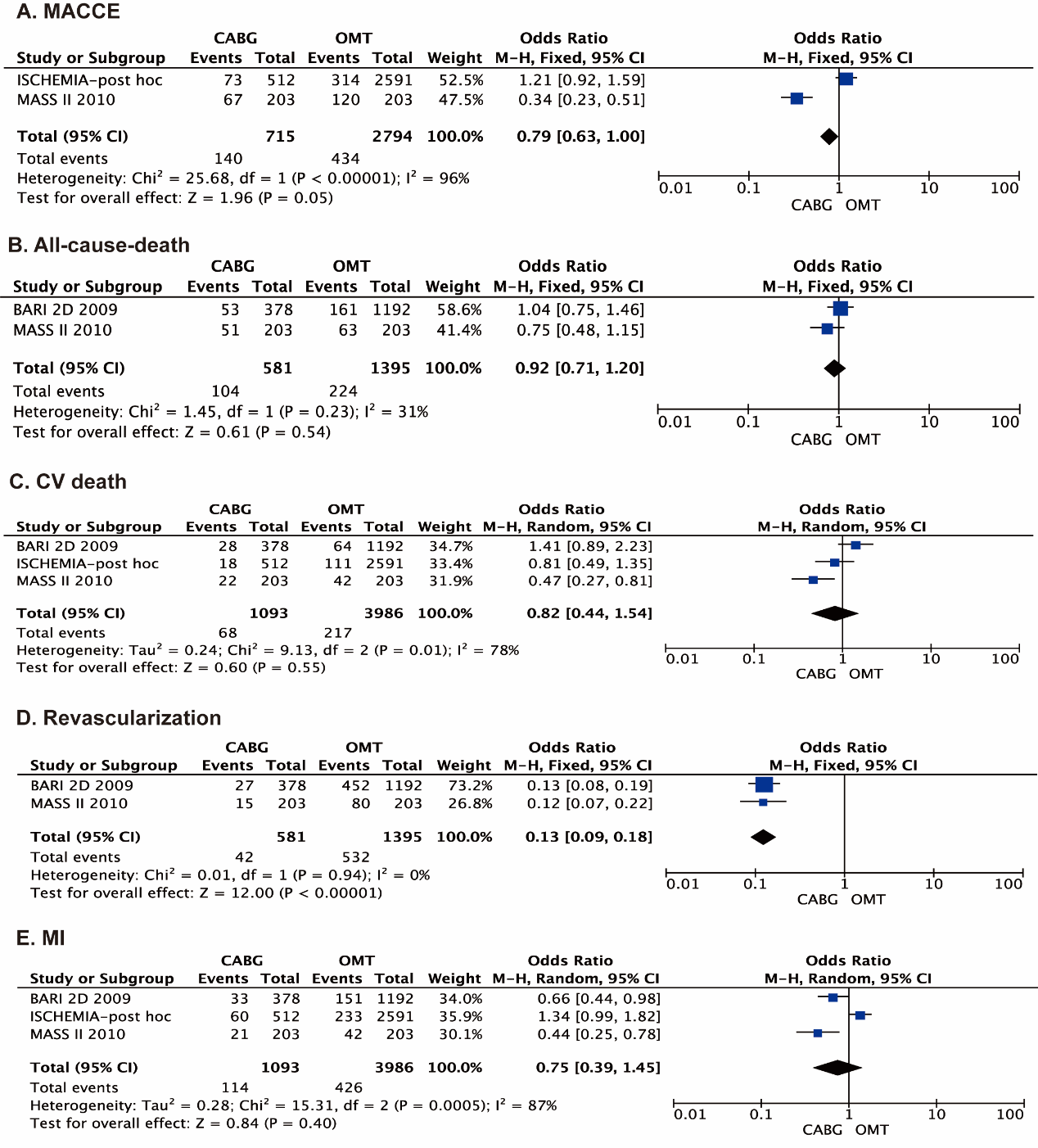
Figure S2: Subgroup analysis of efficacy outcomes between CABG and OMT.**

**
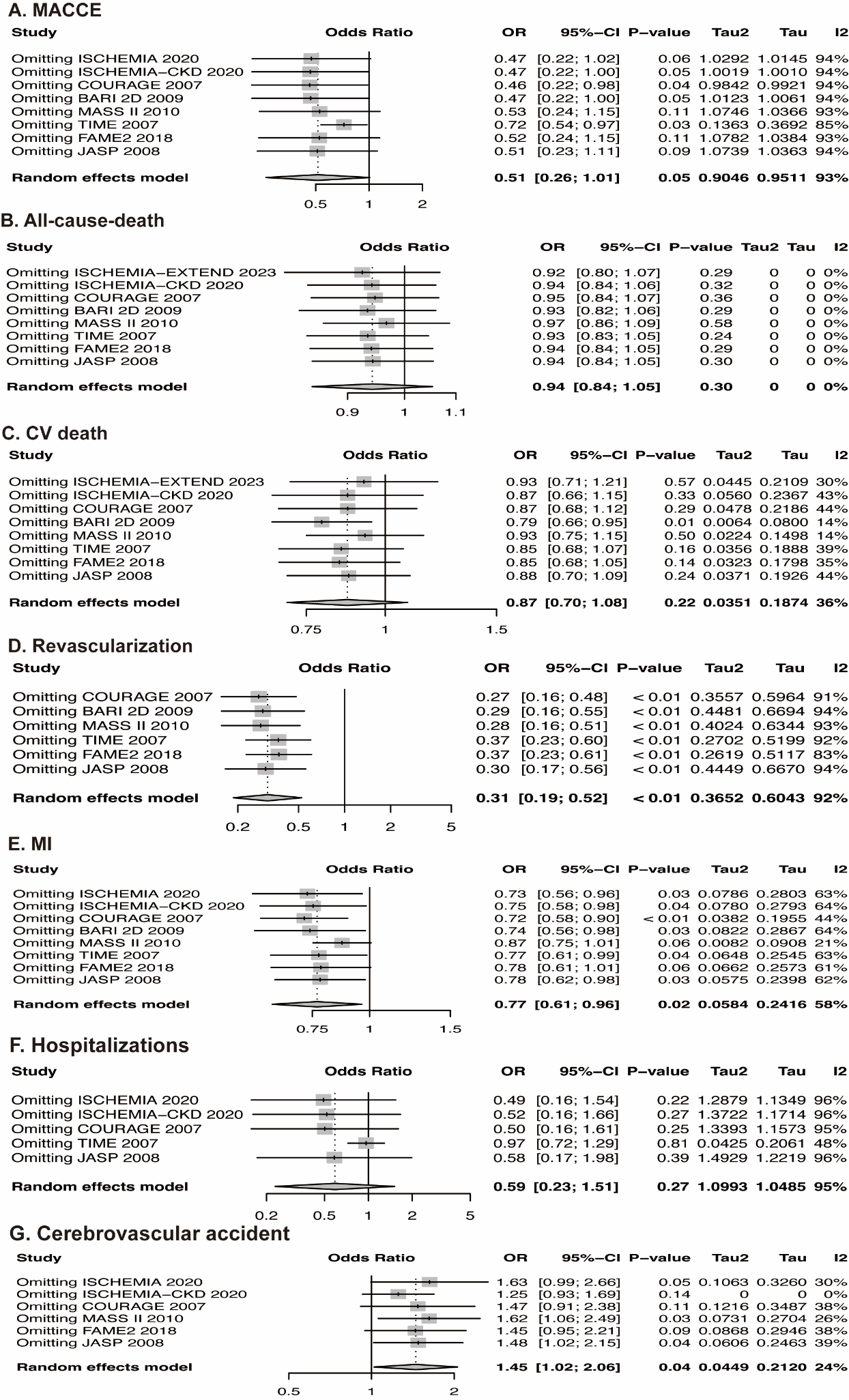
Figure S3: Sensitivity analysis of the efficacy outcomes comparisons.**

**Figure S4: Risk of bias and quality assessment of outcomes**

**
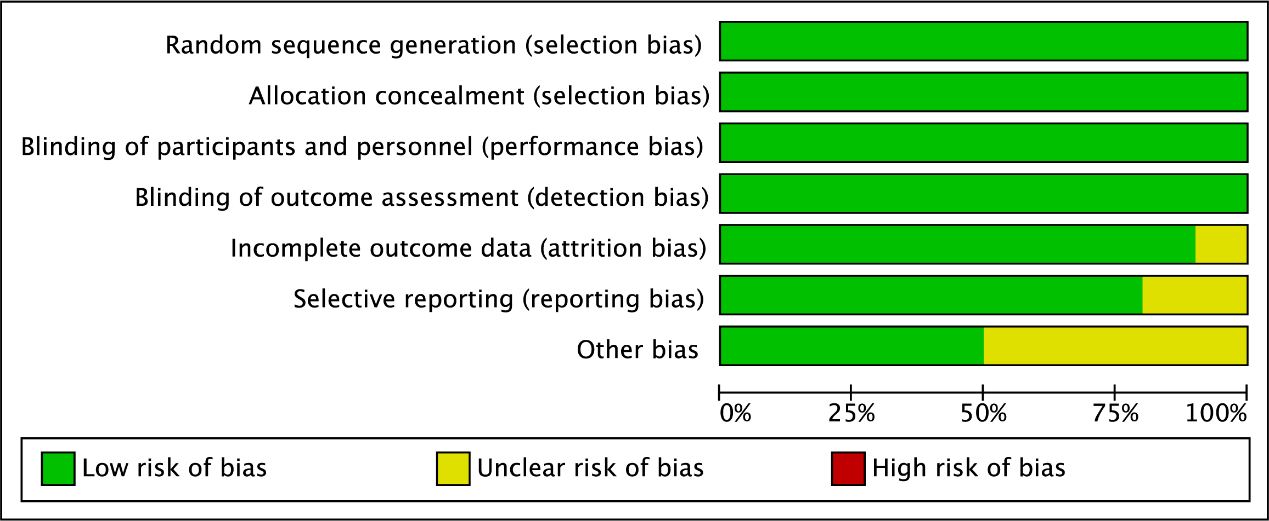
**
